# Successful Long Term Eradication of Factor VIII Inhibitor in Patients with Acquired Haemophilia A in Saudi Arabia

**DOI:** 10.4084/MJHID.2012.021

**Published:** 2012-04-02

**Authors:** Galila Zaher, Soheir Adam

**Affiliations:** King Abdulaziz University

## Abstract

Acquired haemophilia A is a serious and potentially fatal bleeding disorder. Diagnosis is difficult and maybe delayed due to its rarity. The high mortality rate and the complex nature of treatment necessitate patient management at a haemophilia centre, where the required expertise and resources are available. Prompt diagnosis is crucial and early initiation of therapy could be life saving. Management includes initial control of bleeding followed by an approach to eradicate the coagulation factor inhibitor. In this paper we describe our local experience with acquired haemophilia A, which resulted in the successful control of major bleeding at presentation and eradication of inhibitors.

## Introduction

Acquired haemophilia A (AHA) is a rare but often fatal bleeding disorder.^1^ Patients typically lack previous or family history of bleeding. Presentation is acute with sudden onset of bleeding, spontaneously or following a hemostatic challenge. In contrast to congenital haemophilia, which is characterized by hemarthrosis, bleeding in patients with AHA commonly affects soft tissue.^2^ AHA is associated with a high mortality rate of 9–22% if untreated.^2^ Prompt diagnosis and management are important for a favorable outcome.

In this paper we describe our local experience with idiopathic AHA demonstrating the heterogeneity of clinical presentation, prognostic and therapeutic aspects of this disorder.

## Definition

AHA is caused by the development of auto-antibodies against coagulation factor VIII. ^[2]^ Although the most common acquired coagulation factor inhibitors are those directed against FVIII, inhibitors to other coagulation factors, including factor V, and factor IX have also been described.^3^ (Table 1)

## Genetic basis

**Figure 1:**
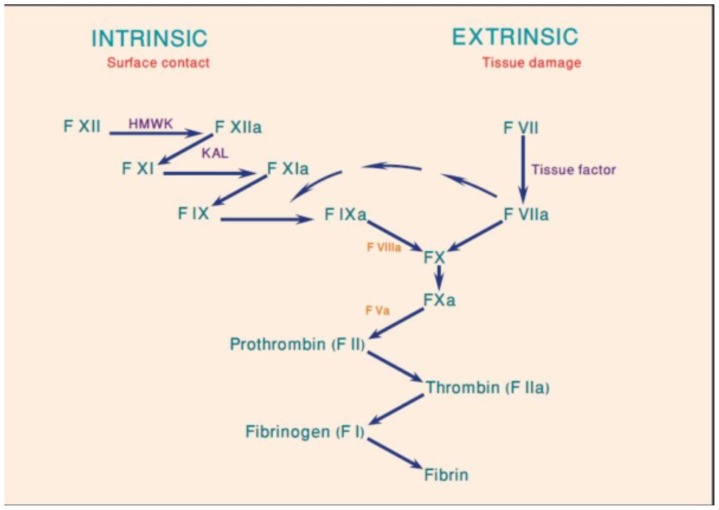
The coagulation cascade

Factor VIII is synthesized as a 330-KDa-precursor protein with an A1-a1-A2-a2-B-a3-A3-C1-C2 domain structure.^4^ After proteolytic processing, FVIII associates with von Willebrand Factor (VWF) in heterodimers. FVIII functions as a cofactor to factor IXa in the tenase complex (Figure 1). FVIII inhibitors are primarily oligoclonal or polyclonal immunoglobulins G (IgG1 or IgG4). These antibodies are non-complement fixing and non-precipitating immunoglobulins that bind FVIII in a time-and temperature-dependent manner. Most FVIII inhibitors bind to A2, A3 or C2 domains.^5^ Anti-C2 domain antibodies disrupt the FVII binding site to both phospholipid and VWF, while antibodies to A2 and A3 domains interfere with FVIII binding to FX and FIXa. In contrast allo-antibodies in congenital haemophilia A, where antibodies inactivate FVIII in first-order kinetics, the auto-antibodies in AHA tend to show a rapid initial inactivation phase followed by a slower phase of equilibrium in a non-linear inactivation pattern or type II kinetics where some factor VIII can usually be measured.^6^ The complex type II kinetics make it difficult to evaluate the clinical importance of the inhibitor titer level or the factor level. Recent findings suggest that polymorphism in immune regulatory genes are associated with the incidence of AHA. The polymorphic genetic profiles of these genes differ between ethnic groups and may partly explain the variation observed in different population.^7^

## Incidence

The estimated incidence of AHA varies (0.1–1.5 per million/population per year)^1^,^2^ with a median age at presentation of 65 years.^8^

## Underlying Diseases

Nearly fifty to sixty percent of AHA cases occur spontaneously particularly in elderly individuals.^9^ Therefore most inhibitors are labeled as idiopathic, yet several other series and case reports have suggested an underlying pathology (Table 2).^2^ The most commonly associated conditions are autoimmune disorders (17%), malignancies (15%), pregnancy (7–21%), drugs hypersensitivity and infections. Autoimmune disorders like systemic lupus erythematosus. Sjogen syndrome, and rheumatoid arthritis have been associated with AHA.^10^–^13^ Other frequently reported co-morbidities are malignancies including liquid and solid tumors. Acquired haemophilia is commonly associated with solid tumors more than lymphoproliferative diseases.^14^–^17^ Drugs including penicillin and interferon have also been reportedly associated with AHA.^18^–^19^

The postpartum period is one of the more frequent settings in which AHA may occur therefore it should be considered early in the evaluation of unusual postpartum bleeding.^20^–^22^ Inhibitors can develop during pregnancy or labor leading to postpartum bleeding and even requiring hysterectomy. Recurrences in subsequent pregnancies has been reported.^22^

## Clinical Presentation

Acquired hemophilia is characterized by lack of past medical or family history of bleeding tendency.^10^ There is no gender difference except in the younger age group because of its the association with pregnancy.^20^–^22^ The majority of patients with AHA present with mucocutaneous type of bleeding including; skin hematomas, epistaxis, ecchymoses, menorrhagia, gastrointestinal and urological bleeding. Hemarthrosis, typically seen in congenital haemophilia A is not a feature of AHA.^3^,^24^ Other manifestations include; excessive bleeding following trauma or surgery and occasionally cerebral hemorrhage.^25^

## Diagnosis

**Figure 2:**
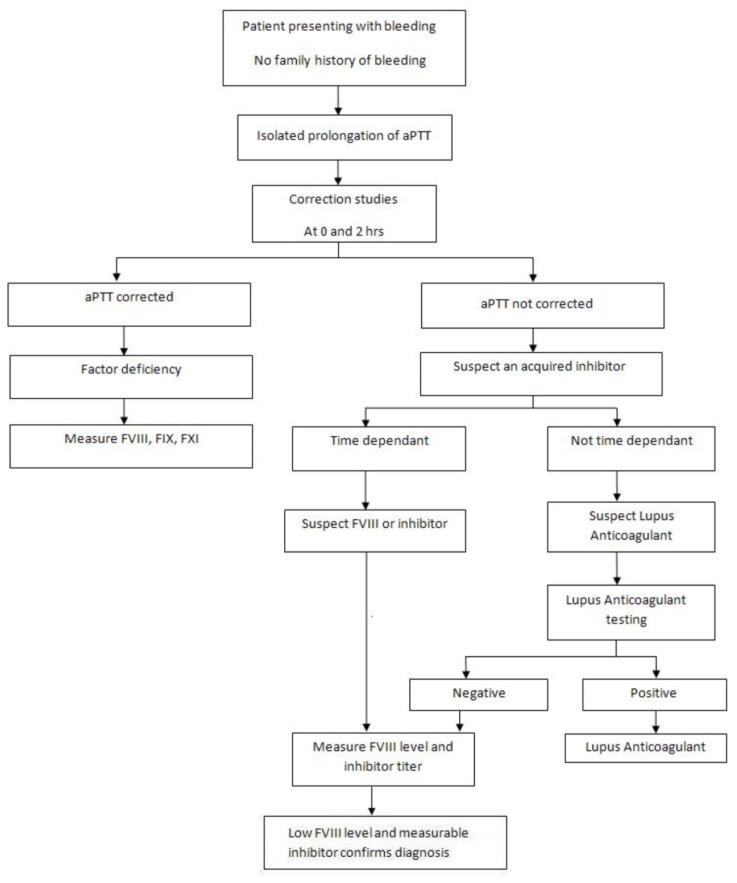
Diagnostic approach to a bleeding patient

Frequent delays in the diagnosis of AHA may occur owing to the rarity of the disorder and absence of family history of bleeding.^2^ A structured approach to diagnosis should be implemented to ensure prompt initiation of treatment. (Figure 2) The combination of an isolated prolongation of aPTT with a normal platelet count, prothrombin time (PT) and thrombin time (TT) in a patient with acute bleeding, should raise the suspicion of a coagulation factor inhibitor. Failure to correct the prolonged aPTT after mixing with normal plasma at a 1:1 proportion after incubation for 2 hours at 37^0^C favors the presence of inhibitor rather than factor deficiency.^27^ An APTT prolongation of 10–15 second longer than the control is strongly suggestive of the presence of inhibitors. Furthermore, the characteristic time-depended nature of FVIII antibodies confirms their presence.^28^–^32^ Factor VIII activity level should be checked and the antibody titer should be quantified using the Bethesda assay.^32^,^33^ The residual FVIII activity after incubation of normal plasma with serial dilutions of patient plasma for 2 hours at 37^0^C is measured to quantify inhibitor titer. One Bethesda unit (BU) per mL is the amount of inhibitor needed to inactivate 50% of factor activity in one milliliter of normal plasma. The Nijmegen modification adds buffer to minimize shifts in pH.^33^,^34^

## Methods

Data were collected from patient’s medical records after obtaining an informed consent and approval of the hospital Ethical Committee. Blood samples were collected and processed according to international standards.^29^,^32^

## Case 1

### Clinical presentation

A 15-years-old girl presented to our hospital on the 31^st^ of March, 2002 with hematochezia, melena and menorrhagia. The patient denied any past history of bleeding and her family history was unremarkable for bleeding disorder. She was not receiving any medications and had no history of recent illness.

### Investigation

Laboratory tests revealed normocytic, normochromic anemia (haemoglobin 6.3 g/dL), and isolated prolongation of aPTT (118 seconds/control 31 seconds). Fibrinogen, PT, and platelet count were all within normal range. The aPTT failed to correct with normal plasma indicating the presence of inhibitor. She had a low factor VIII activity (FVIII: <1 IU/L), and a normal von Willebrand factor (VWF) level. Family study revealed normal FVIII and VWF level in both parents, which excludes true haemophilia in a female patient. The inhibitor titer was >500 Bethesda units (BU/mL). Investigations for conditions most frequently associated with the formation of inhibitors; like autoimmune disorders, malignancies and dermatological disorders were all negative. Both Upper and lower gastro-intestinal endoscopies revealed no local causes of bleeding.

### Management

The patient was started on recombinant FVII (rFVII) (Novoseven®, Novo Nordisk, Denmark) 90 mg/kg, intravenous tranexamic acid 1gm 8 hourly and received 2 units of packed red blood cells (PRBC). Immune suppression by cyclophosphamide (100 mg/m^2^) and oral prednisolone (1 mg/kg) was started after counseling and consent of her parents. The patient received a second dose of rFVIIa (Novoseven®, Novo Nordisk, Denmark) and her clinical picture improved objectively over the following days as evidenced by the cessation of gastrointestinal bleeding. The patient was discharged on cyclophosphamide and tapering doses of prednisone.

### Follow-up

When next seen in clinic, patient reported no bleeding manifestations and her menstrual cycle was regular. During follow up the patient was given a single dose of rVIIa (Novoseven®, Novo Nordisk, Denmark) 90 μg/kg before a scheduled dental extraction. Extraction was performed successfully and the patient had no excessive bleeding following the procedure. Laboratory tests showed aPTT of 40 seconds, FVIII level of 80 IU/dL and an inhibitor titer zero BU/mL. Her parents had concerns about the carcinogenic potential of cyclophosphamide so it was discontinued after completing three month of the treatment. The patient was followed up for 9 years with no evidence of recurrence.

## Case 2

### Clinical presentation

A 77-years-old male presented to the emergency room with a massive muscle hematoma in the right gluteal region following an intramuscular injection on the 5^th^ of March, 2006. He had a past medical history of diabetes, hypertension and chronic obstructive pulmonary disease. His medication list included an oral hypoglycemic agent (Glucophage) and antihypertensive medications (Amaryl and Captopril).

### Investigation

Initial laboratory investigations showed normocytic, normochromic anemia (hemoglobin 6.5 g/dL) and markedly prolonged aPTT (116 seconds/control 32 seconds). PT, fibrinogen and platelet count were all within normal ranges. The absence of any previous history of bleeding suggested an acquired hemophilia, which was confirmed by failure of APTT to correct after mixing with normal plasma following 2 hours of incubation at 37^0^C. Plasma F VIII level was very low (F VIII <I IU/dL) and the FVIII inhibitor titre was 64 BU/mL.

### Management

Despite the high antibody titer, the patient had mild bleeding which did not present a life-threatening situation; therefore no systemic hemostatic agents were utilized. He received 2 units of packed red blood cell. Cyclophosphamide 100mg/m^2^ orally was started, however steroids were not given because of comorbid uncontrolled hypertension and diabetes. Anti-phospholipid antibodies including lupus anticoagulant, anticardiolipin antibody (IgG and IgM) and B2 glycoprotein antibodies (IgG and IgM) were all negative. Computed Tomography (CT) scan of the chest and abdomen showed a left anterior chest wall collection but there was no evidence of an underlying malignancy. The hematoma gradually resolved and the patient was discharged on cyclophosphamide and did not require further transfusions upon follow up.

### Follow-up

when seen in cinic 3 months later, the patient had no bleeding and no treatment- related toxicitities. The aPTT, and FVIII level were both within normal range and FVIII inhibitor was absent. Tests for antinuclear antibodies remained weekly positive (titer 1:80). Cyclophosphamide was thus discontinued and the patient was followed up for 4 years with no evidence of recurrence.

## Case 3

### Clinical presentation

A 59-year old male presented to the emergency department complaining of tongue bleeding and submental swelling on the 5/1/2009. He had a history of non-insulin dependent diabetes mellitus and benign prostatic hypertrophy and he was not on any medication. There was no family history of bleeding.

### Investigations

the patient had severe normocytic normochromic anemia (hemoglobin 6.1 g/dL) and an isolated prolongation of aPTT (88 seconds/control 32 seconds). All other coagulation tests were normal. The aPTT failed to correct upon mixing with normal plasma. Factor VIII level was very low (<1 IU/dL) and FVIII inhibitor level was 83 BU/mL.

### Management

on the second day of admission, the swelling progressed to the submandibular area with the development of dyspnea, dysphagia, hoarseness of voice and stridor. The ENT team performed nasal fibro-optic endoscopy, which revealed a hematoma of the tonsils and epiglottis. The patient was immediately transferred to intensive care unit (ICU) and started on rFVIIa (Novoseven®, Novo Nordisk, Denmark) 90μg/kg intravenous bolus dose. Dexamethasone (8 mg IV twice daily) and tranexamic acid (1 gm IV every 8 hours) were started and four units of packed red blood cells were transfused. A second dose of rFVIIa was required 3 hours later to stop the bleeding. The submandibular swelling subsided, and the patient regained his voice. He was discharged on day 7 on prednisolone 1mg/kg and cyclophosphamide 100 mg/m^2^ orally. The search for an underlying disorder did not identify a culprit.

### Follow-up

Upon follow-up in the hematology clinic 6 weeks later, his aPTT was 31.1 seconds, F VIII was 80 IU/dL and FVIII inhibitor was undetectable. He continued on cyclophosphamide and prednisone taper. Both medications were discontinued after 6 months of treatment and the patient was followed up for 2 years and remained in haematological remission. Laboratory data are presented in Table 3.

## Discussion

These cases confirm the heterogeneity of AHA from the etiologic and clinical points of view. Meta-analysis of retrospective and prospective surveys on patients with AHA identified three prognostic factors independently associated with a decreased overall survival.^1^,^36^ These factors were: age, related conditions (e.g. malignancy, post-partum period and others), and achievement of complete remission. Our cases are in the line with this evidence; in fact all patients were diagnosed as idiopathic AHA and they achieved a complete remission and had a long-term survival. Cases 2 and 3 were elderly, which is inparallel with the median age of presentation. Case 2 had borderline positive test for antinuclear, antibodies, but had no underlying autoimmune disorder. The appearance of a coagulation factor inhibitor may herald the clinical onset of on autoimmune disease by several years. However the patient was followed up for 6 years, which favors the diagnosis of idiopathic AHA. Novoseven® was used safely and effectively for management of severe bleeding episode in two cases. Tranexamic acid proved effective in cases I and 3 as an adjuvant therapy for treating hemorrhage in areas with high fibrinolytic activity, like the oral cavity and the uterus. All cases responded favorably to the combination of steroids and cyclophosphamide as first line treatment for inhibitor eradication.

Case 1 was a young female who, due to the potential risk of secondary leukaemia associated with cyclophosphamide, she elected to discontinue it. Rituximab would have been a good option for her, but it was not available at the time of her initial presentation. Despite old age of presentation in two of the patients, which is usually associated with a poorer prognosis, these patients attained and sustained complete remissions. This highlights the importance of epitope characterization in the pathogenesis of AHA. All three patients are alive up to the time of writing this report. One patient developed an elevated blood sugar level and worsening control of previously diagnosed non-insulin dependent diabetes mellitus related to prednisone therapy. No neutropenia, sepsis, thrombocytopenia or other treatment-related complications have been observed.

## Management

In contrast to congenital haemophilia, there are no randomized studies on the treatment options for patients with AHA, which leaves the treatment decisions to the discretion of the treating physician. Management of AHA should be undertaken at a haemophilia center where sufficient experience is available.^1^ Bleeding-related mortality rate is high approaching 15%–20%,^30^ which emphasizes the importance of early diagnosis and prompt treatment. The aims of the treatment are to control bleeding and to eventually eradicate the antibody. A conservative approach and close observation and follow up of the titer for 4–6 weeks may be appropriate in children, pregnancy and drug-associated AHA and in patients with low titer presenting with minor bleeding.^27^,^28^ Management of acute bleeding should be started promptly and in a step-wise fashion. Once patient is stabilized, immunosuppressive therapy should be started to eradicate the inhibitor. Data on inhibitor eradication, however, are based on relatively small, uncontrolled single-center cohorts and a meta-analysis.^35^–^37^

The selection of a treatment option depends on age, underlying disorders, bleeding site and severity as well as inhibitor level.^29^,^31^ Normal haemostasis can be achieved by two main mechanisms; use of haemostatic agents, or FVIII concentrates. Haemostatic agents include Recombinant FVII, aPCC, 1-deamino-8-D-argininevasopressin DDAVP and antifibrinolytic agents. Clinical studies have examined the efficacy of treatment options and good hameostatic efficacy has been demonstrated for recombinant activated factor VII (rVIIa) and factor VIII inhibitor bypass activity (FEIBA).

## Correction of FVIII Level

Correction of FVIII could be achieved by human plasma-derived, recombinant FVIII concentrate or (DDAVP).^37^–^39^ Human FVIII concentrate is the treatment of choice for patients with persistently low inhibitor titers (<5 BU/mL) presenting with minor bleeds, yet the response is unpredictable. The aim of the treatment in patients with minor bleed is to keep plasma levels of FVIII of 30–50%.^28^,^40^ It may be advantageous in some cases to use FVIII concentrate that also contains von Willebrand factor (VWF). For a newly diagnosed patient with a moderate or severe bleeding, a high bolus dose of 100–200 IU FVIII/kg body weight may be used. Factor level should be determined at least once daily to monitor the laboratory response. However; the clinical response is a better guidance in AHA.

## Haemostatic Agents

Deamino-8-D-arginine vasopressin (DDAVP) infusion can provide a rapid, albeit transient rise in factor VIII levels in patients with very low titer inhibitors, which could be sufficient to treat minor bleeding.^38^,^39^ However, DDAVP treatment alone is insufficient to maintain hemostasis in most patients. Tachyphylaxis limits the use of DDAVP on consecutive days and its antidiuretic and vasomotor side effects should be carefully considered in children and older patients.^40^

In patients with high titer (>10 BU/mL) or those presenting life or limb threatening bleeding factor VIII infusion is often unsuccessful.^28^,^40^ These patients are generally treated with inhibitor by pass therapy. Patients who fail to respond to bypass therapy should be tried on plasmapheresis^41^ and immune-suppressive medications^42^ or with immunoadsorption.^43^,^44^ Plasmapheresis alone or in addition to immunoadsorption of FVIII antibodies using staphylococcal protein A may be useful for patients with high titer inhibitors with severe bleeding or in preparation for surgery who have failed to respond to bypassing agents. Factor VIII replacement is needed immediately after plasmapheresis to achieve hemeostasis.

## By Passing the Inhibitor

By passing the inhibitor can be achieved using activated prothrombin complex or recombinant activated Factor VII. Factor eight by passing agent (FEIBA) is a virally inactivated, plasma-derived concentrate of activated clotting factors. The mechanism of action of FEIBA as a bypassing concentrate is not fully understood. [45] Retrospective studies have shown that hemostasis can be achieved with FEIBA in 76% of cases at a dose of 75units/kg, 8 hourly.^46^,^47^ Clinical response is used to monitor treatment due to lack of a suitable method for laboratory monitoring.^40^ A total dose of 200 units/kg should not be exceeded within a 24-hour period because of the risk of thromboembolic complications. [48] The concomitant use of FEIBA with systemic tranexamic increases the risk of thrombosis and is therefore not recommended.^40^ Lack of clinical improvement is an indication for switching to other by passing agents.^29^,^40^

Recombinant activated FVIIa binds to tissue factor (TF) released at the site of tissue injury resulting in thrombin generation thus bypassing the need for FVIII.^49^,^50^ The European medicines Agency (EMEA) concluded that rFVIIa’s benefits are great for the treatment and prevention of bleeding episodes in patients with congenital haemophilia with inhibitors undergoing surgery, congenital factor VII deficiency and Glanzmann’s thrombasthenia. Recombinant FVII is given at a dose of 60–120 mg/kg. Summer et al reviewed 139 patients with AHA treated with rFVII with an overall clinical response of 74%. [50] Thromboembolic events have been previously reported following the use of rFVII.^51^,^52^

## Therapy to Eradicate FVIII Inhibitors

Although spontaneous remission may occasionally occur, most published guidelines and treatment algorithms recommend autoantibody eradication once the diagnosis is made, since bleeding complications can prove to be fatal.^29^,^40^ Approaches to eradicate inhibitors include; immunosuppression, intravenous immunoglobulin (IVIG) and plasmapheresis. Immunosuppressive therapy is indicated for idiopathic, autoimmune and malignancy related cases. Data in inhibitor eradication, however, are based on relatively small, uncontrolled single-centered cohorts and a meta-analysis. Most centers have adopted a treatment regimen consisting of corticosteroids, cytotoxic drugs, or a combination of the two, originally studied by Green & Lechner.^10^ The prednisone, alone or in combination with cyclophosphamide, azathioprine, cyclosporine or 2-chlorodeoxyadenosine is recommended as first line treatment.^29^,^40^ Prednisone at a dose of 1 mg/kg per day for a minimum of six weeks followed by a rapid taper of the dose, eradicates the inhibitor in 1/3 of the patients.^30^ The estimated median time to remission is 39 days for steroids alone however, relapse is not uncommon (20%) when immunosuppression is stopped or reduced, requiring the administration of a second course.^30^ Failure to respond to corticosteroids after a period of 2 to 3 weeks calls for an alternative innumosuppressive regimen, like cyclophosphamide^52^–^55^ or rituximab or both. The addition of cyclophosphamide at a dose of 1.5–2 mg/kg per day at maximum 3–4 months can increase the response rate to 60–80%.^30^,^55^ Cyclophosphamide is best avoided in women or men in the child bearing age, since it has a teratogenic and carcinogenic effect. [56,57] Monitoring of side effects such as: leucopenia, thrombocytopenia and anemia is recommended. The estimated median time to remission is about 49 days for combination therapy.^30^ Rituximab is a chimeric monoclonal antibody which targets the CD20 antigen on the B-cell lymphocytes. It induces apoptosis primarily in pre B-cell clones and has an immunosuppressive mode of action.^58^ The off-label use of rituximab has been studied in AHA, at a weekly dose of 375 mg/m^2^ IV for 4 consecutive weeks showed promising results with durable remission particularly in patients with low titers.^58^–^60^ Rituximab alone or in combination with other modalities is considered as a second-line therapy if first-line therapy fails.^29^

The exact mechanism of action of intravenous immunoglobulins (IVIG) is not fully understood, however possible mechanisms proposed include neutralization caused by the presence of anti-idiotype antibodies, suppression of inhibitor production and temporary displacement of inhibitors. A total dose of 2 g/kg over 2 or 5 days induced complete or partial remission in 30% of patients particularly those with low titers.^62^ Multiple courses are needed to obtain a sustained response. The high cost of this treatment makes it a reasonable option as a second line therapy for those patients who fail to respond to immunosuppression. The value of adding IVIG to immunosuppressive medication to improve the chances of inhibitor eradication is controversial.^30^

## Immune Tolerance Induction

Immune tolerance is an accepted and effective treatment in patients with congenital haemophilia with inhibitor but has rarely been applied in AHA. Complete and sustained remission was achieved in more than 90% of the patients.^63^ The combination of Malmo and Bonn protocol (MBMP) which includes immunoadsorption and inhibitor elimination, factor VIII substitution, IVIG and immunosuppression can reduce the mean time to achieve cessation of bleeding to 3 days with MBMP protocol compared to 3 weeks for various conventional treatment regimes.^64^ Complete remission of 88% was reported with this protocol.

## Prognosis

Patients with AHA have a high mortality rate 9–22%. Patients in whom the inhibitor could not be eliminated had a higher mortality. Factors predicting a favorable response to immunosuppressive therapy are low inhibitor titers and short interval between the appearance of the inhibitor and initiating therapy.^30^ Low titer inhibitors especially in the early stages are more likely to disappear.^30^ Younger age is associated with better survival and better eradication of inhibitor, while older age is associated with poorer outcome and a higher mortality rate.^36^ The best prognosis is seen with inhibitors developing during the postpartum period, with favorable outcomes reported in 40–97% of patients.^36^ Eradication of the inhibtor associated with malignancy is not always possible, but treatment of the tumor can accelerate inhibitor eradication.^17^,^36^ The relapse rate after CR is about 20%,^30^ and median time to relapse is around 7 month after cessation of therapy.^30^,^40^ Advanced age, presence of underlying malignancy and failure to attain a CR were shown to independently affect the mortality.^36^

## Conclusion

Patients with AHA represent a demanding clinical challenge. The morbidity and mortality are high, and treatment involves the use of specific and expensive coagulation promoting products. The treatment of the patients with AHA should ideally be at a haemophilia center with expertise in this area. No standard treatment protocol has been defined for the treatment of acute bleeding episodes nor for inhibitor eradication and therapy often needs to be individually tailored. FVIII inhibitors in the cases discussed here were totally eradicated with no recurrence despite long term follow up, which is unusual for AHA. We believe that environmental and genetic factors may play role in the heterogeneity of the disease. Further studies can help identify the unique characteristics and epidemiology of AHA in Saudi Arabia.

## Figures and Tables

**Table 1:** Coagulation factor inhibitors and associated disorders

Target Coagulation Factor	Associated Disorders
V	Lymphoproliferative disorders, adenocarcinoma, tuberculosis, aminoglycosides, topical thrombin
IX	Systemic lupus erythematosus, acute rheumatic fever, hepatitis, collagen, vascular diseases, multiple scierosis, postprostatectomy, and postpartum
XI	Autoimmune diseases, prostate carcinoma, chronic lymphocytic leukemia, chlorpromazine
XIII	Idiopathic, isoniazed, penicillin
VWF±	Autoimmune disorders, monoclonal gammopathies, lymphoproliferative diseases, epidermoid malignancies, hypothyroidism, myeloproliferative disorders, and certain medication
II	Topical thrombin, idiopathic, autoimmune diseases, procainamide
VII	Bronchogenic carcinoma, idiopathic
X	Amyloidosis, carcinoma, acute nonlymphocytic leukemia, acute respiratory infections, fungicide exposure, idiopathic

**Table 2:** Underlying pathology associated with acquired hemophilia A

Disease Association	Green 1981	Collins 2007
Idiopathic	46.1**%**	63.3**%**
Collagen, vascular, and other autoimmune diseases	18.0	16.7
Solid and hematological Malignancies	6.7	14.7
Dermatological Disease	4.5	3.3
Possible drug reaction	5.6	NR
Pregnancy and Postpartum Period	7.3	2.0
Other (infections, vaccinations)	11.8	NR

**Table 3:** Laboratory values for patients before and after treatment

Case	1		2		3	
	Before	After	Before	After	Before	After
PT Sec	12	12	13	12.5	12.4	12.2
aPTT Sec	218	92	126	36	88	31
FVIII	<1U/dL	79 U/dL	<1U/dL	87<U/dL	<1U/dL	80<U/dL
Bethesala BU/ml	500	0	64	0	83	0
